# Commercialism in Dentistry

**Published:** 1916-09

**Authors:** 


### COMMERCIALISM IN DENTISTRY.

Frequently writers in our dental journals grow enthusiastic in describing the efforts of a few practitioners who lay claim to uniqueness in that they do not hesitate to spend days or even weeks in performing operations ordinarily taking so many minutes or hours to complete. All the devices known to modern dentistry are requisitioned and made to pay tribute. Time and expense are obviously of little account, provided desirable results are obtained.

Such men are working under ideal conditions, we say, and we envy them this distinction. Seldom, if ever, are we advised as to the amount of their remuneration. This would be a mixing of the sublime with the ridiculous, and as such can not be entertained. "There are no wealthy dentists"—such was often told us when we were students with a yearning to follow in the footsteps of the illustrious. "The only dentists who even approach the status of 'wealth' are those who have adopted unscrupulous methods in their practices"—this is a view that has even found expression in our dental literature.

The effect of such teachings—one is almost inclined to call them farcial teachings—is to engender in the minds of dentists a disregard for the monetary side of his labors. What a sad thing it is to see some members of our profession who, in their earlier years, gave unstintingly of their time and skill in advancing the cause of dentistry and are now without means of support. They are dependent upon others for a livelihood. A pitiful spectacle, we say, and yet it ought to serve as an object lesson. There are two sides even to a professional career—service and reward. One is just as important as the other.

Recognizing that a "laborer is worthy of his hire" it does not seem fair that we should charge as "commercial" those members of our profession who, while seeking new and better methods of dentistry, gather to themselves what is at best only a fair return for their labors. A dentist does not necessarily become "commercial" because of giving up his private practice to take part in the production of some article for the use of the profession. Such men deserve our help and sympathy and should not be discouraged by reason of our unjust censure. Dentistry needs, more than anything else, men who will do research work. Let us see that we survey the ground carefully before condemning any of our associates.—*Oral Health.*

